# Clot in Transit: Therapy via Peripherally Inserted Central Catheter Line

**DOI:** 10.7759/cureus.21691

**Published:** 2022-01-28

**Authors:** Ala Mustafa, Jacob Obholz, Mostafa Ghanim, Samuel Congello

**Affiliations:** 1 Internal Medicine, MercyOne North Iowa Medical Center, Mason City, USA; 2 Cardiology, MercyOne North Iowa Medical Center, Mason City, USA

**Keywords:** thrombolysis, pulmonary embolism, picc line, catheter directed therapy, clot in transit

## Abstract

There are currently no definitive guidelines for the optimal management of clots in transit (CIT) due to a distinct lack of quality research to suggest a recommended therapy. The three main treatment modalities that are commonly utilized for pulmonary emboli (PE) (a sequela of CIT) are thrombolysis, pulmonary embolectomy, and anticoagulation alone. The current recommendation for severe PE with hemodynamic collapse is to consult cardiothoracic surgery for clot retrieval. One ongoing area of research involves the use of catheter-directed application of thrombolytic agents as it may have similar outcomes to the systemic application while minimizing the risk of bleeding events due to a lower dose of medication used. We report the case of a patient in whom, by taking advantage of an already placed peripherally inserted central catheter (PICC) line, tissue plasminogen activator (tPA) was successfully delivered at a localized site near the clot for active thrombolysis while only causing minimal adverse effects related to recent laminectomy/fasciectomy and foraminotomy compared to what may have been observed with systemic tPA administration.

## Introduction

Clots in transit (CIT) are an uncommon life-threatening medical emergency. CIT is generally defined as a right heart thrombus that is unattached to any intracardiac structure. Thus, it is effectively a mobile thrombus waiting to embolize. It is usually visualized and diagnosed with echocardiography to help differentiate between a right heart thrombi that may have resulted from atrial fibrillation and those originating from deep venous thrombosis. CIT is likely underdiagnosed, with a current estimated incidence of around 4-18% in patients suffering from pulmonary embolism (PE) and is a medical emergency with reported in-hospital mortality rates as high as 44.7% [1,2]. Currently, there is no ideal treatment for the condition; however, surgical embolectomy, intravenous (IV) thrombolysis, catheter-directed thrombolysis, or anticoagulation are often utilized [1]. In this report, we present the case of right atrial thrombus and bilateral PE with evidence of right heart strain in a patient recently hospitalized for bilateral C4-5 decompression, laminectomy/fasciectomy, and foraminectomies. Given the risks presented in this case due to recent spinal surgery, a joint decision by cardiology, cardiothoracic surgery, and neurosurgery was made to treat the patient with catheter-directed thrombolytic therapy.

## Case presentation

**Figure 1:**
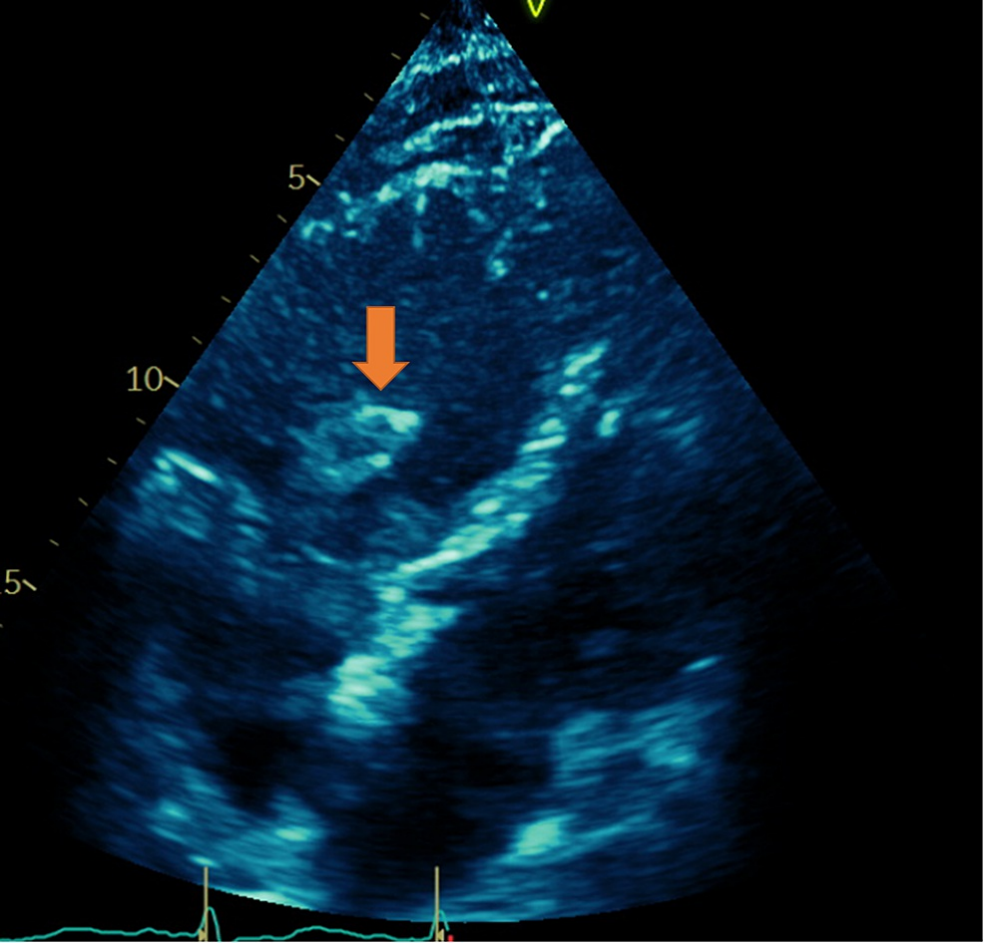
Four-chamber view demonstrating clot burden within the right atrium (arrow)

**Figure 2:**
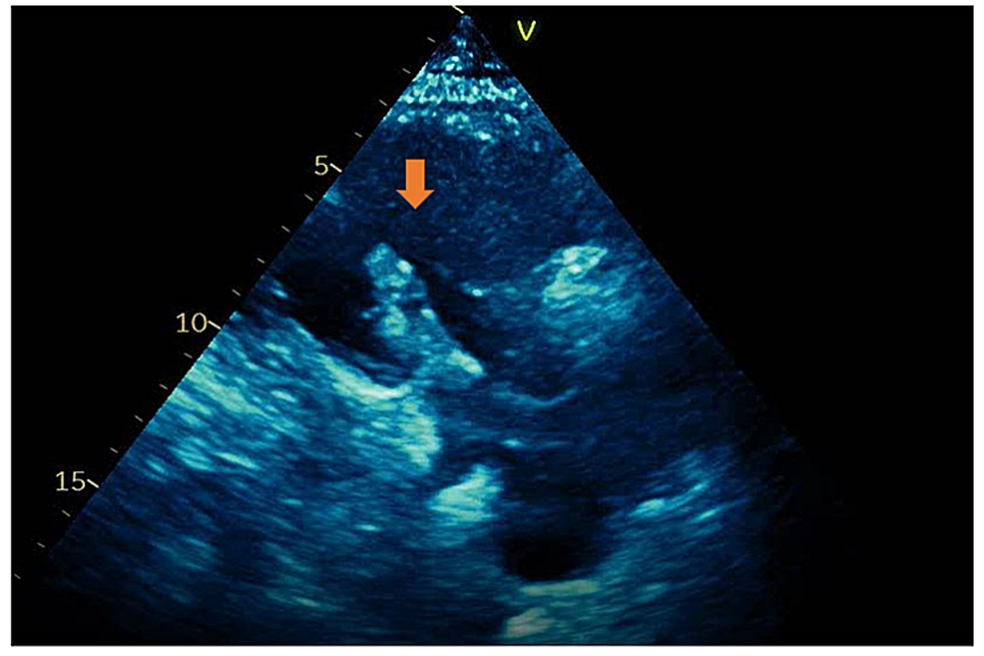
Right ventricle inflow view showing large mobile thrombus crossing the tricuspid valve (arrow)

An 81-year-old man presented with a past medical history of coronary artery disease status post coronary artery bypass graft (CABG) for triple vessel disease, hypertension, type 2 diabetes, obstructive sleep apnea, and history of panhypopituitary syndrome secondary to pituitary tumor removal. The patient was admitted to the hospital due to progressive lower extremity weakness after undergoing an L3-5 decompressive laminectomy one month prior. The decision was made by the neurosurgery team to perform L5-S1 laminectomy and medial facetectomy bilaterally, which helped resolve the patient's initial acute complaint. Further evaluation revealed that the patient also suffered from cervical myelopathy. Six days later, the patient underwent C3-5 laminectomy and medial facetectomy with decompression bilaterally. He was not placed on deep vein thrombosis prophylaxis immediately following his surgery out of concern for bleeding risk. The patient’s postoperative course was initially uneventful; however, two days after his cervical decompression, the patient suddenly developed hypotension with readings ranging from 62/43 to 77/42 mmHg, dyspnea requiring 2-L supplemental oxygen via nasal cannula, and EKG findings consistent with right bundle branch block. An echocardiogram was performed and showed a mobile mass in the right atrium (Figures 1, 2).

**Figure 3:**
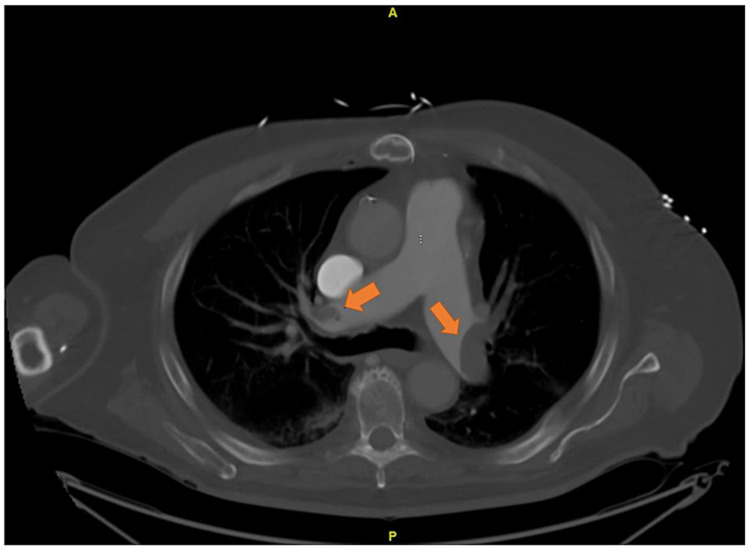
CT of the chest showing large bilateral clot burden (arrows) CT: computed tomography

No evidence of deep vein thrombosis was appreciated on imaging or physical exam. The patient was transferred to the ICU, where he was started on norepinephrine at 2 mcg/min for vasopressor support. At this time, neurosurgery was updated, and cardiology was also consulted, who jointly decided to start a high-intensity unfractionated heparin infusion (with a partial thromboplastin time goal of 70-101.9 seconds) until further investigations were performed. Additionally, a CT of the chest was performed, which showed bilateral pulmonary emboli with large clot burden with right heart strain and a clot measuring 5-6 cm within the right atrium (Figure 3).

The patient was felt to have a CIT and high-risk PE with a Pulmonary Embolism Severity Index (PESI) score of 143. He was then evaluated by the cardiothoracic surgery team for clot removal; however, due to the patient's history of CABG, a repeat sternotomy would have led to a very high risk of injuring a patent left internal mammary artery. Unfortunately, percutaneous aspiration techniques were also not available at the facility during the time of presentation. Due to the large clot in the right atrium and fear of dislodging with further hemodynamic collapse, it was decided through shared decision-making with the patient to give tissue plasminogen activator (tPA) therapy via a previously placed peripherally inserted central catheter (PICC) line with entry at the origin of the right atrium to save the patient from another potentially complicated procedure to replace the catheter.

**Figure 4:**
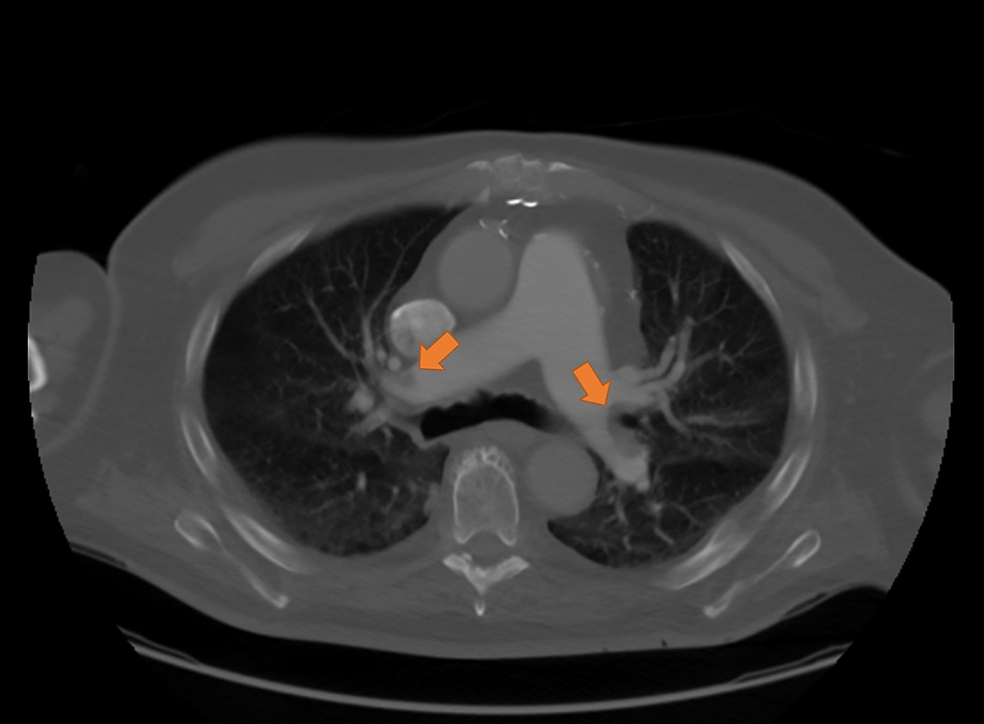
CT chest following tPA therapy showing reduced clot burden compared to the previous exam (arrows) CT: computed tomography; tPA: tissue plasminogen activator

**Figure 5:**
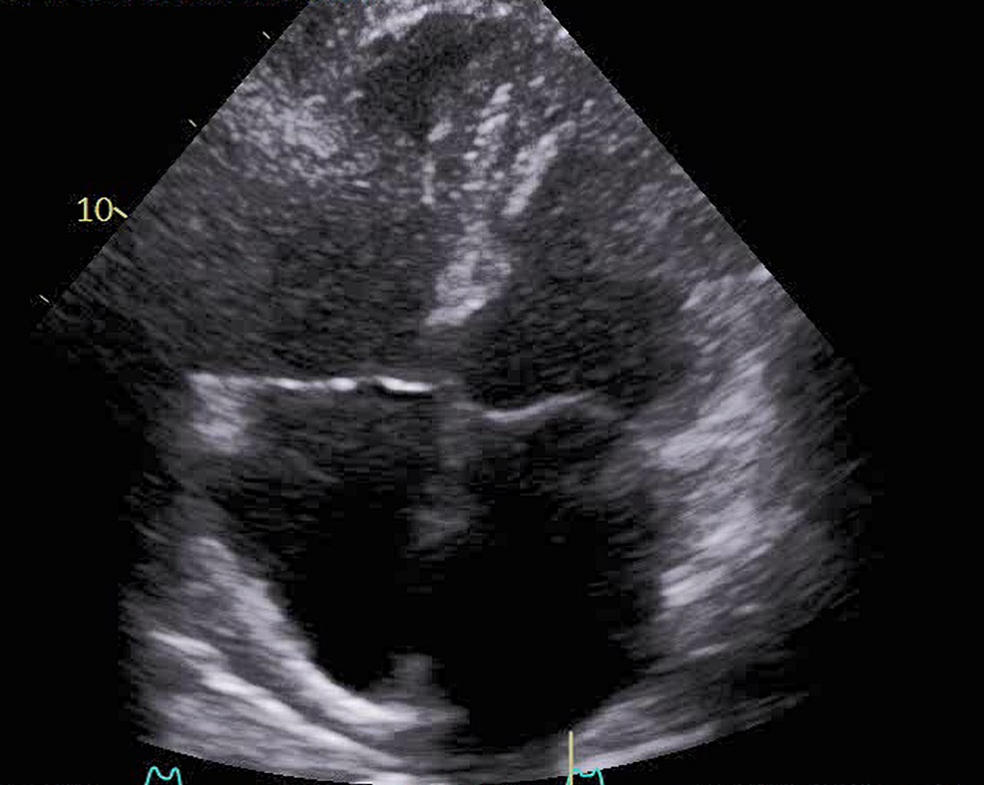
Limited echocardiogram following tPA therapy showing complete resolution of the intracardiac mass tPA: tissue plasminogen activator

The patient was given a 2,000-unit bolus of heparin and started on an infusion rate of 1,400 units/hr. He was then started on alteplase 1 mg/hr for a total of 14 hours. The following day, six hours after the discontinuation of the tPA and anticoagulation, the patient's cervical wound was inspected and noted to have increased in firmness and ecchymosis, which raised suspicion for underlying hematoma. No airway compromise was noted from the ongoing bleed, but there were some negative changes to the patient’s upper extremity strength bilaterally. The patient was taken back to the operating room by the neurosurgery team for hematoma evacuation with drain placement. No other immediate complications were noted. A follow-up CT scan of the chest revealed that the sagittal PE was slightly reduced in volume from the prior exam, but the right heart enlargement and interventricular septal bowing were resolved (Figure 4). Limited echo showed the complete resolution of the intracardiac mass (Figure 5). Given the patient's normal estimated creatine clearance adjusted for ideal body weight (>60 mL/min), he was started on dabigatran 150 mg twice daily for continued outpatient anticoagulation as there was an available reversal agent if required. He subsequently made a full recovery after being discharged back to his assisted living facility.

## Discussion

CIT of the right heart is a potentially life-threatening complication that, unlike other clot disorders such as deep vein thrombosis, cannot be treated with standard anticoagulation therapy. As mentioned previously, there are limited options for treating this condition and it requires a case-specific approach as noted here. Our patient, due to his history of previous CABG, was not a candidate for surgical approach and required tPA, despite undergoing recent neurosurgery. Catheter-directed thrombolysis was chosen with the intent to minimalize the risk of complications that could have been observed with higher doses of tPA. In this case, a PICC line was already in place, which was utilized to deliver the medication. The patient did develop a hematoma, which required surgical drainage. Although minor, the risk of fatal embolization far outweighed the risk of bleeding.

When CIT is coupled with intermediate-risk PE, there is a very high mortality rate with no morbidity benefit when comparing treatment choices. Recent studies on CIT report mortality rates associated with various methods as follows: no therapy, anticoagulation therapy, surgical embolectomy, and thrombolysis at 100%, 28.6%, 23.8%, and 11.3% respectively [3]. Additionally, there has been no observable difference in long-term mortality among patients with intermediate-risk pulmonary emboli when comparing thrombolysis and anticoagulation alone as most deaths occur within 30 days of the original event [4]. Systemic thrombolysis rapidly decreases the strain on the right heart due to clot burden, but at the cost of an increased risk of major bleeding [5]. However, there has been no evidence thus far suggesting that early thrombolysis improves patients’ functional status, disease burden, or cardiac recovery as measured with imaging modalities such as echocardiogram [4].

Many of the treatment guidelines for blood clots have been developed around the risk stratification of PE, but there exists a special consideration for PE secondary to CIT versus that with other causes. The severity of PE due to CIT is often underestimated due to a lack of clinical findings such as hemodynamic instability [6,7]. Systemic thrombolysis is the recommended therapy for high-risk PE. Surgical- or catheter-directed embolectomy can be considered; however, in our patient, it was felt that those were not valid options. Eventually, the delivery of thrombolysis through the PICC line resolved CIT and relieved hemodynamic compromise due to the large central pulmonary emboli. This approach was technically easy to apply and did not require central venous access cannulation of the heart or central pulmonary artery, thereby decreasing the risk of clot infiltration or vascular injury. Thus, this was a successful case of the resolution of a CIT in combination with high-risk PE with an improvement of hemodynamic status via PICC line-directed thrombolysis. The consideration of this for therapy for high-intermediate-risk or high-risk PE to avoid systemic thrombolysis or pulmonary artery cannulation in the absence of any significant intracardiac shunt on echo is very promising and thought-provoking. Further studies are needed to develop an appropriate risk stratification algorithm in order to determine which treatment option is best suited for individual patients while also assessing the risk of mortality and hemorrhage.

## Conclusions

This case highlights the successful treatment of a massive PE with associated CIT using catheter-directed thrombolytic therapy through a PICC line. Currently, there are no definitive guidelines for the optimal management of CIT owing to a distinct lack of quality research to recommend any particular therapy. Often, a selective approach must be taken by a multidisciplinary team along with shared decision-making with the patient to choose the best therapy available for a specific patient. Due to multiple complicating factors, it was deemed appropriate to attempt thrombolytic therapy through a previously placed PICC line in this scenario. Although there was a significant complication of a cervical hematoma, it could have been much worse given that the nature of such bleeds is often impossible to predict. The patient ultimately recovered, indicating that catheter-directed thrombolysis through a PICC line may be a valid approach in unique situations.
